# Deep learning-based vessel segmentation in non-contrast black-blood MRI for treated aneurysm follow-up: a comparative study with TOF-MRA and DSA

**DOI:** 10.1007/s00234-026-03936-7

**Published:** 2026-03-06

**Authors:** Samer Elsheikh, Alexander Rau, Petra Cimflova, Urs Würtemberger, Elias Kellner, Horst Urbach, Marco Reisert

**Affiliations:** 1 Department of Neuroradiology, University Medical Center Freiburg, Freiburg, Germany; 2 Department of Diagnostic and Interventional Neuroradiology, University Hospital of Bern, Bern, Switzerland; 3 Medical Physics, Department of Diagnostic and Interventional Radiology, University Medical Center Freiburg, Freiburg, Germany; 4 Department of Stereotactic and Functional Neurosurgery, University Medical Center Freiburg, Freiburg, Germany

**Keywords:** Automated vessel segmentation, Intracranial aneurysm recurrence, Black-blood MRI (BB-MRI), Time-of-flight MRA (TOF-MRA), Digital subtraction angiography (DSA), Endovascular treatment follow-up

## Abstract

**Introduction:**

Automated segmentation of intracranial vessels in 3D black-blood T1W-MRI is feasible. This study evaluates its utility for detecting recurrent aneurysms and compares automatically labeled MRI (AL-BB-MRI) with time-of-flight MRA (TOF-MRA) and digital subtraction angiography (DSA).

**Materials (patients) and methods:**

For model development, the basal intracranial arteries were manually labeled in 37 3D black-blood T1W-MRI examinations from 31 patients with previously endovascularly treated aneurysms. An independent, consecutive series of 84 patients with 90 aneurysms was assessed by three readers to identify recurrences in AL-BB-MRI and TOF-MRA. DSA served as the reference standard when available.

**Results:**

The model achieved adequate vessel segmentation (Dice = 0.81). Agreement between AL-BB-MRI and TOF-MRA was in 70/90 (78%) aneurysms. Major recurrence rates were similar: 16/90 (18%) aneurysms on AL-BB-MRI and in 14/90 (16%) aneurysms on TOF-MRA. Discrepancies were most common in anterior communicating aneurysms (seven seen only in AL-BB-MRI) and basilar artery aneurysms (five only in TOF-MRA). Compared to DSA (*n* = 22), AL-BB-MRI had similar accuracy (73% vs. 77%), sensitivity (62% each), and specificity (79% vs. 86%). AL-BB-MRI and TOF disagreed in nine aneurysms: AL-BB-MRI was false positive in three and false negative in two, while TOF-MRA was false positive in two and false negative in another two aneurysms.

**Conclusions:**

AL-BB-MRI showed high agreement with TOF-MRA in recurrence detection of aneurysms in the anterior circulation. Both modalities performed similarly against DSA. AL-BB-MRI was superior after flow-diverter or stent-assisted treatment but less reliable for intrasaccular flow disruption. (Level of Evidence: 5B).

The study was registered in the German Clinical Trials Register (DRKS00014644) on 07.05.2018.

**Supplementary Information:**

The online version contains supplementary material available at 10.1007/s00234-026-03936-7.

## Introduction

Following endovascular treatment (EVT), approximately 25% of cerebral aneurysms recur and 9% require retreatment [1]. While TOF-MRA is the clinical standard for recurrence detection, it is technically limited by flow artifacts and signal loss [2–5]. Signal degradation worsens after flow diverter placement due to susceptibility artifacts and is most severe following intrasaccular flow disruption, where susceptibility artifacts combine with the Faraday cage effect [6, 7].

As an alternative, high-resolution 3D black-blood T1-weighted MRI is commonly used in neurovascular imaging. These sequences provide excellent depiction of the vessel lumen and vessel wall morphology [8] and are widely applied in vessel wall imaging as well as for assessing post-EVT aneurysm enhancement, while overcoming the signal loss associated with flow diverters [8–13].

To effectively interpret this 3D data, optimal visualization is essential. The cerebral vascular tree is a complex 3D structure that is difficult to assess in source images alone. Consequently, 3D visualization is a favored evaluation method [14], as reflected in the routine clinical use maximum intensity projections for TOF-MRA assessment.

Convolutional neural networks (CNNs) enable precise segmentation of vascular structures in medical images [15]. However, prior cerebrovascular CNN studies have primarily used TOF- or contrast-enhanced MRA [16], with only one employing 3D black-blood T1W-MRI [17].

Therefore, we developed a CNN for cerebral vessel segmentation on non-contrast 3D black-blood T1W-MRI. This study aimed to evaluate whether automatically labelled black-blood MRI (AL-BB-MRI) provides noninferior diagnostic performance for detecting post-EVT aneurysm recurrence compared to the clinical standard, TOF-MRA, using DSA as the reference standard.

## Materials and methods

This prospective study enrolled consecutive patients undergoing post-EVT intracranial aneurysm follow-up between May 2017 - March 2023. Our institutional follow-up protocol for endovascularly treated intracranial aneurysms uses TOF-MRA as the primary screening modality. Digital subtraction angiography is additionally performed as a baseline examination at the first follow-up for all cases involving flow diverters or intrasaccular flow disruptors, and for retreatment candidates with large recurrences identified on TOF-MRA during follow-up. Regular screening examinations are scheduled at 6, 18, 36, and 60 months, then at 3- to 5-year intervals after treatment. For cases with questionable or small recurrences, we schedule earlier follow-up TOF-MRA. Inclusion criteria in this study were age ≥ 18 years and clinically indicated post EVT follow-up with MRI and/or DSA.

### Human ethics and consent to participate

The Institutional Review Board approved the protocol in accordance with the Declaration of Helsinki (approval number: 12/17). All participants provided written informed consent prior to inclusion.

### Imaging techniques

All MRI scans were acquired on a 3 T Siemens Magnetom Prisma (Erlangen, Germany) with a 64-channel head coil. The protocol included 3D black-blood T1W-MRI for analysis and TOF-MRA as the recurrence reference standard (scan parameters in Table 1). Corresponding DSA studies, performed within 100 days of MRI on a Philips Allura FD20/20 biplane system (Best, the Netherlands), were included for comparison. Image processing used the Nora platform (https://www.nora-imaging.com).

**Table 1:** Scan parameters of the study sequences

**Variable**	**3D black-blood T1W-MRI**	**TOF-MRA**
Orientation	Saggital	Axial
TR, TE, FA, NEX, PAT	800, 10, N/A, 1, GRAPPA 2. echo train length 269 ms	21, 3.43, 18, 1, GRAPPA 2
Field of View	225 x 225 mm	200 x 200 mm
Matrix	384 x 384	346 x 384
Slice Thickness	0.64 mm	0.6 mm
Slices	256	168
Voxel Size	0.55 x 0.55 x 0.60	0.52 x 0.52 x 0.60
Scan Duration	6:54 min	5:36 min

TOF-MRA, time-of-flight magnetic resonance angiography; FA, flip angle; GRAPPA, generalized autocalibrating partially parallel acquisition; PAT, parallel acquisition technique.

### Imaging data partitioning

**Fig. 1:**
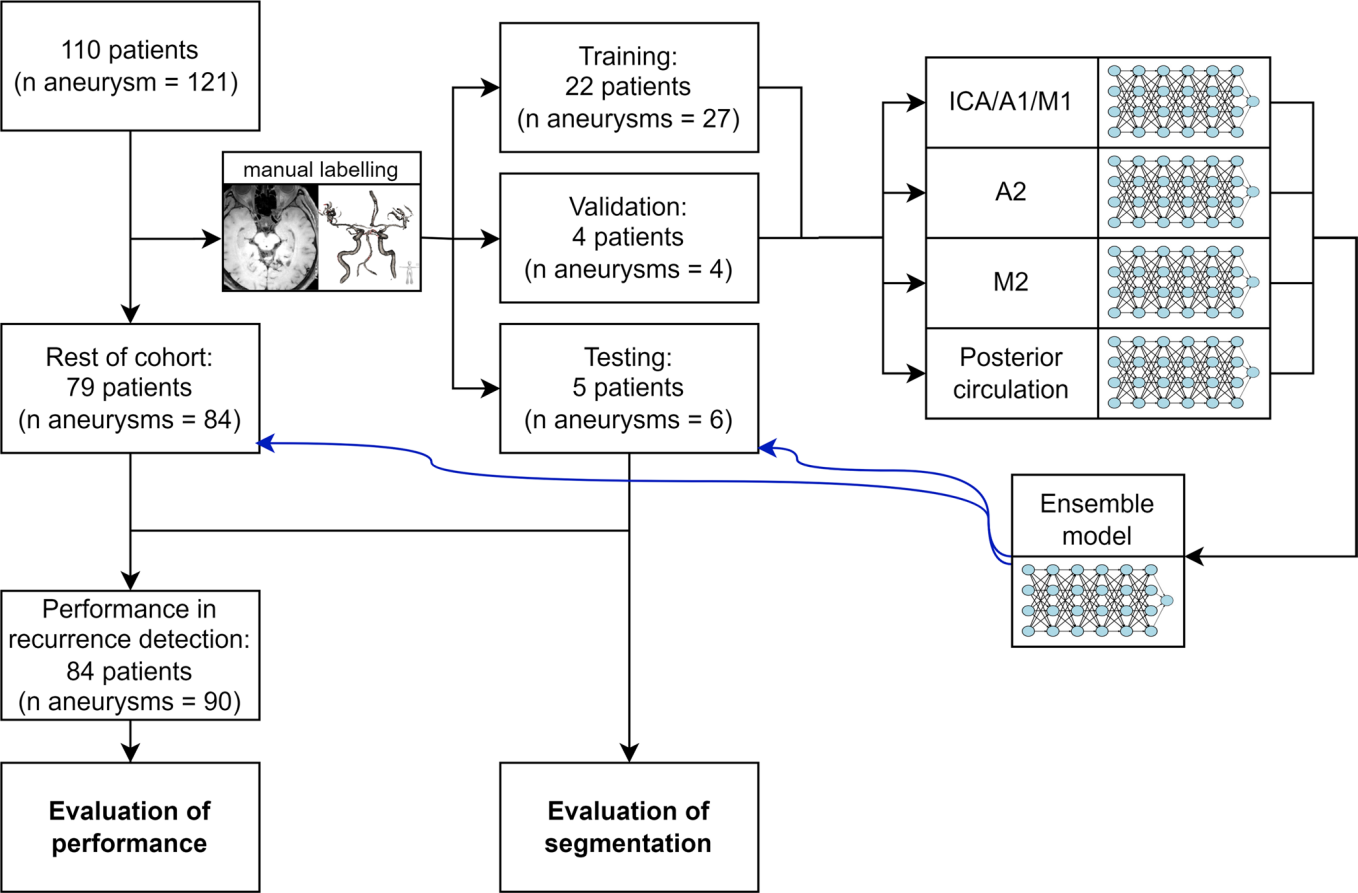
Flow chart of patient enrollment and grouping as well as a schematic depiction of the methods

The study included 110 consecutive patients with 121 treated aneurysms. For CNN development and testing, we used 37 examinations from 31 randomly selected patients, partitioned on the patient level and randomly assigned into: training (*n* = 27 scans/22 patients), validation for (*n* = 4 scans/4 patients) and testing (*n* = 6 scans/5 patients) groups for training, hyperparameter tuning and reporting of evaluation metrics, respectively. Recurrence detection evaluation involved 84 consecutive patients (aneurysm *n* = 90), incorporating the testing group, undergoing their first post-EVT MRI follow-up (Fig. 1).

## Model development

The ROI covered common aneurysm locations. Based on a prior publication demonstrating suboptimal performance in smaller vessels [17], we created region-specific models for vascular territories with comparable vessel calibers:

1. ICA, M1-segment, A1-segment and anterior communicating artery.
2. A2-segment of the anterior cerebral artery.
3. M2-segment of the MCA.
4. Posterior circulation, including intracranial vertebral, basilar, and posterior cerebral arteries.

Binary masks were manually generated in Montreal Neurological Institute space (Supplemental Data Figure S1) and transformed to patient space using SPM12 (Wellcome Centre for Human Neuroimaging, London, United Kingdom). A senior neuroradiologist (S.E., 20 years’ experience) annotated intracranial arteries on 37 3D black-blood T1W-MRI scans from 31 patients.

All CNN models were developed using the Patchwork CNN Toolbox [18], implementing a hierarchical, multiscale network with a U-Net + + architecture in each scale [19]. This architecture featured nested and dense skip connections, as well as intermediate convolutional blocks, between the down-sampling and up-scaling arms, with loss function calculation at each up-scaling node. Training was performed with a fixed learning rate of 0.001 using the Adam optimizer, batch size of 32, with random weight initialization and batch normalization applied throughout. No dropout layers were used, and no early stopping strategy was applied.

For training the four subregional models, 3D black-blood T1W-MRI served as input. Each model produced a 3D NIfTI output containing per-voxel probabilities of vascular tree membership, maintaining native resolution and patient coordinate space. This allowed direct overlay of the output on the source images and 3D surface rendering. The probabilistic outputs from all subregional models and the original 3D black-blood T1W-MRI served as input for an ensemble model to produce the final predictions.

Model parameters included: number of scales, 3; Top-k loss, set to 1000. Augmentation in left-right, anteroposterior and superior-inferior directions: random rotation of φ = 0.2, random flipping and random scaling of 0.2. A U-Net + + architecture; network depth of 6 having the following feature dimensions [24, 32, 48, 64, 128, 256], giving 1.97 × 10^7^ trainable and 15,456 non-trainable parameters for each model. We trained the model in iterations, each iteration included 64 patches from each image and trained for 4 epochs. Model training was stopped after 1 × 10^7^ patches.

### Aneurysm recurrence

The primary outcome measure, aneurysm recurrence, was evaluated in AL-BB-MRI and TOF-MRA by three radiologists (P. C., A. R., and U. W., with 11, 6, and 8 years of neuroradiological experience, respectively) using a binary scale (no/minor < 3 mm vs. major recurrence) [20]. Assessments occurred in separate sessions ≥ two months apart, with readers blinded to clinical data (except aneurysm location).

The presentation included 3D multiplanar reconstructions of TOF-MRA and 3D black-blood T1W-MRI with AL-BB-MRI superimposed on the 3D black-blood T1W-MRI as an overlay and additionally shown as a 3D surface render. Discrepancies were resolved using a majority vote. All DSA results were evaluated by the first author using a binary adaptation of the Raymond-Roy scale [21].

### Statistical evaluation

For statistical analysis, R statistical software v4.4.0 was employed [22].

For evaluation of segmentation models, overlap and spatial distance metrics were calculated using the DeepMind library (https://github.com/deepmind/surface-distance), with a primary focus on the Dice Similarity Coefficient (DSC).

For evaluation of recurrence detection performance of AL-BB-MRI compared to TOF-MRA, absolute percentage agreement was calculated between modalities. While TOF-MRA is widely used for aneurysm recurrence detection, it is not the gold standard. We compared TOF-MRA and AL-BB-MRI using agreement measures rather than accuracy. Due to expected class imbalance (9% major recurrence prevalence [1]), we used Gwet’s AC1 or Fleiss’ κ (as appropriate) instead of Cohen’s κ to avoid the “Kappa paradox” [23, 24].

Agreement measures included:

1. Reader performance: Individual vs. other readers and vs. consensus to identify outliers.
2. Modality interpretability: Interrater agreement within each modality.
3. AL-BB-MRI vs. TOF-MRA: Intrarater and consensus agreement.

For evaluation of consensus readings of AL-BB-MRI and TOF-MRA with DSA, we reported accuracy, sensitivity, and specificity [25].

Subgroup analyses examined:

1. exact anatomical location.
2. vascular territory (ICA, anterior cerebral artery, middle cerebral artery, or posterior circulation territories).
3. implanted device (flow diverter, intrasaccular flow disruption or stent-assisted coiling).

## Results

The mean age of our cohort was 54.58 (± 11.84) years and females constituted 63.16% (*n* = 72). Table 2 presents per-group patient demographic data, treatment methods, and results (further per-group details are available in Supplemental Data Table S1).

**Table 2:** Patient characteristics and treatment modalities and results in all datasets

**Variable**	**Training (22 patients 27 aneurysms)**	**Validation (4 patients 4 aneurysms)**	**Testing (5 patients 6 aneurysms)**	Recurrence (84 patients 90 aneurysms)*
Age	55.6 (±13.2)	46.8 (±12.3)	53.5 (±5)	54.3 (±11.7)
No. of female patients	16 (59%)	0 (0%)	2 (33%)	63 (70%)
Presentation: incidental	19 (70%)	1 (25%)	5 (83%)	42 (47%)
Presentation: ruptured	8 (30%)	3 (75%)	1 (17%)	48 (53%)
Treatment methods				
Coiling	22 (81%)	4 (100%)	5 (83%)	78 (87%)
Balloon-assisted coiling	16 (59%)	2 (50%)	5 (83%)	59 (66%)
Stent-assisted coiling	3 (11%)	1 (25%)	0	10 (11%)
Flow diverter	7 (26%)	0	1 (17%)	10 (11%)
Intrasaccular flow disruption	1 (4%)	0	0	7 (8%)
Treatment results				
Complete occlusion	6 (22%)	4 (100%)	5 (83%)	51 (57%)
Neck remnant	11 (41%)	0	0	23 (26%)
Aneurysm rest	10 (37%)	0	1 (17%)	16 (18%)
TOF-MRA results				
No / minor recurrence	20 (74%)	4 (100%)	6 (100%)	76 (84%)
Major recurrence	7 (26%)	0	0	14 (16%)

Age is presented as mean ±SD. Categorical variables are presented as number with percentages in parenthesis. *This column includes patients from the testing group

### Model results

**Fig. 2:**
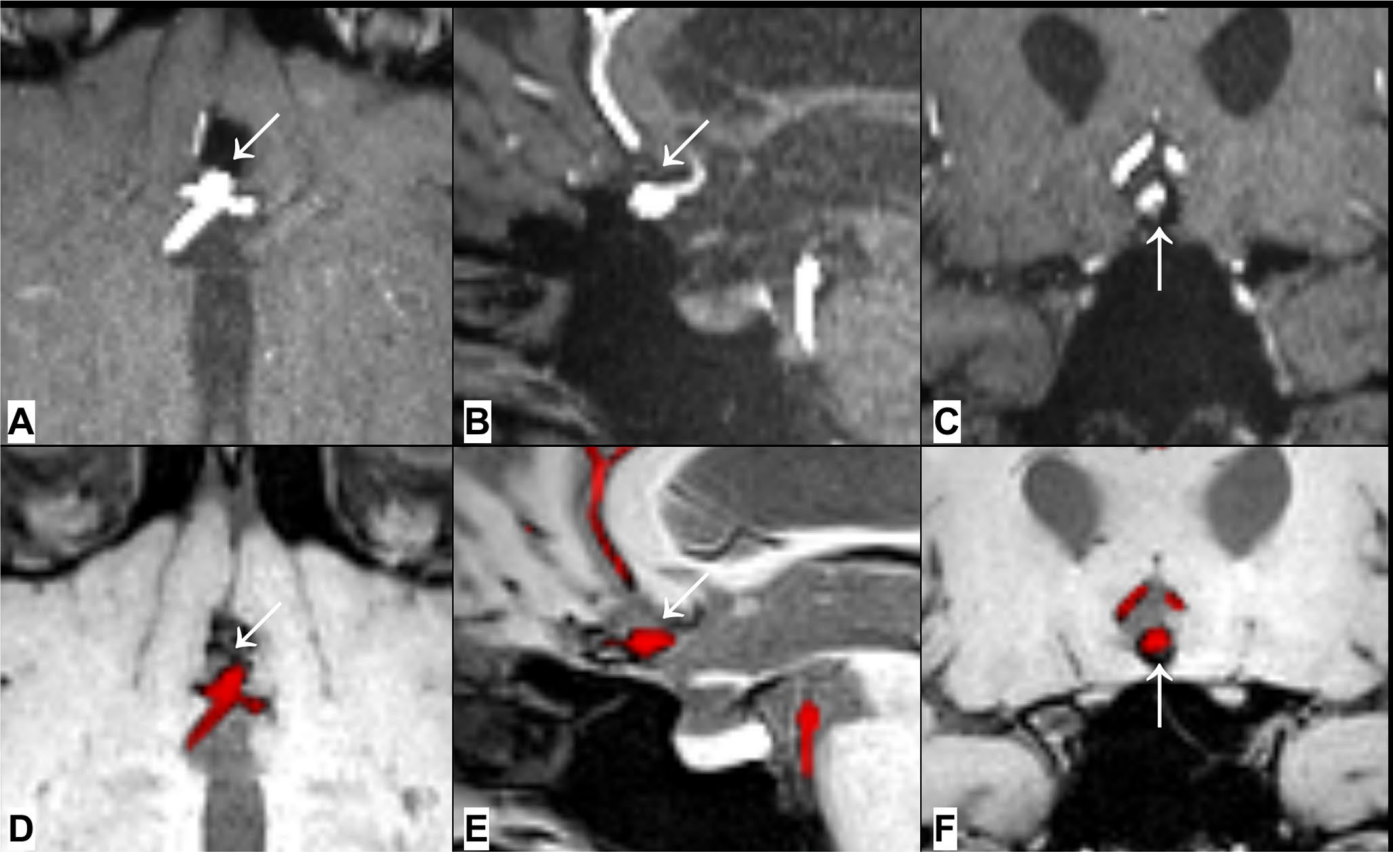
A-C, Axial, sagittal and coronal TOF-MRA multiplanar reconstructions show a major recurrence (arrows) after coiling of an anterior communicating artery aneurysm. D-F, Depict the corresponding segmentation results (red) superimposed on a multiplanar reconstructions of 3D black-blood T1W-MRI, showing the vessels at the base of the aneurysm and the recurrence (arrows)

The subregional models were tested with three loss functions [26]: binary crossentropy and Top-K-Loss (DSC = 0.760 and 0.738 respectively), and focal loss (DSC = 0.694). Top-K-Loss was selected for the final model due to its advantageous performance in the posterior circulation, where patchy artifacts are reported [17]. Model performance metrics are in Table 3 (full metrics in Supplemental Data Table S2), with representative results shown in Fig. 2.

**Table 3:** Metrics of the final models

Model	Dice	Surface dice (1 mm)
A2-segment subregional model	0.67	0.88
ICA/A1-segment subregional model	0.85	0.96
M2-segment subregional model	0.58	0.82
Posterior circulation subregional model	0.73	0.88
Ensemble model	0.81	0.91

Surface dice calculated with tolerance of 1 mm between ground truth and automatically labelled 3D black-blood T1W-MRI.

### Agreement measures of MRI examinations

Consensus readings identified major recurrence in 16/90 (18%) AL-BB-MRI and 14/90 (16%) in TOF-MRA cases (Table 2). Average reader agreement was slightly higher for TOF-MRA (87% pairwise, 93% vs. consensus) than AL-BB-MRI (83% pairwise, 91% vs. consensus). Mean intrarater agreement of 79% and interrater agreement of 74% for AL-BB-MRI and 80% for TOF-MRA. Consensus agreement was 78%. Discrepancies included: AL-BB-MRI detected recurrences missed by TOF-MRA in the anterior communicating artery in seven aneurysms, the basilar artery in three aneurysms, and the posterior communicating artery in one aneurysm, while TOF-MRA detected recurrences missed by AL-BB-MRA in the anterior choroidal artery in five aneurysms, the anterior communicating artery in two aneurysms, the basilar artery in one aneurysm, and the posterior communicating artery in one aneurysm. Detailed agreement statistics are given in Table 4.

**Table 4:** Agreement statistics and subgroup analysis between TOF-MRA and 3D black-blood segmentation

Agreement	Absolute Agreement	Gwets’s AC1 (95% CI)
Mean reader agreement measures		
Pairwise rater: AL-BB-MRI	83%	0.75 (0.61 to 0.88)
Pairwise rater: TOF-MRA	87%	0.82 (0.71 to 0.93)
Single rater vs. consensus: AL-BB-MRI	91%	0.87 (0.78 to 0.97)
Single rater vs. consensus: TOF-MRA	93%	0.91 (0.84 to 0.98)
Agreement measures		
Intrarater: both modalities	79%	0.7 (0.55 to 0.84)
Interrater reading: AL-BB-MRI	74%	0.75 (0.64 to 0.86)
Interrater reading: TOF-MRA	80%	0.82 (0.73 to 0.91)
Consensus reading: both modalities	78%	0.69 (0.55 to 0.84)
Subgroup analysis of consensus reading		
Aneurysm location: paraophthalmic ICA (*n* = 7)	100%	1 (1 to 1)
Aneurysm location: posterior communicating artery (*n* = 12)	83%	0.8 (0.46 to 1)
Aneurysm location: anterior choroidal artery (*n* = 6)	83%	0.8 (0.22 to 1)
Aneurysm location: anterior communicating artery (*n* = 39)	77%	0.65 (0.4 to 0.9)
Aneurysm location: basilar tip (*n* = 12)	50%	0.1 (−0.64 to 0.84)
Vascular territory: anterior cerebral artery (*n* = 41)	78%	0.67 (0.44 to 0.9)
Vascular territory: ICA (*n* = 28)	89%	0.88 (0.73 to 1)
Vascular territory: posterior circulation (*n* = 20)	60%	0.36 (−0.13 to 0.85)
Vascular territory: MCA (*n* = 1)	100%	1 (NaN to NaN)
Flow diverter: no (*n* = 80)	78%	0.68 (0.53 to 0.84)
Flow diverter: yes (*n* = 10)	80%	0.76 (0.32 to 1)
Stent-assisted coiling: no (*n* = 80)	78%	0.68 (0.53 to 0.84)
Stent-assisted coiling: yes (*n* = 10)	80%	0.76 (0.32 to 1)
Intrasaccular flow disruption: no (*n* = 83)	83%	0.78 (0.65 to 0.9)
Intrasaccular flow disruption: yes (*n* = 7)	14%	−0.68 (−1.53 to 0.17)

AL-BB-MRI = Automatically labelled 3D black-blood T1W-MRI

Subgroup analysis of the consensus readings (Table 4) showed:

1. By aneurysm location: 100% agreement for paraophthalmic aneurysms (7/7) vs. 50% for basilar tip aneurysms (6/12).
2. By vascular territory: 89% agreement for ICA (25/28) vs. 60% for posterior circulation (12/20). MCA agreement was in 1/1 aneurysms.
3. By device: 80% agreement after stent-assisted coiling and flow diverter treatment (8/10) vs. 14% after intrasaccular flow disruption (1/7).

### Comparison with DSA

DSA was available for 22 aneurysms (Table 5). True recurrences occurred in eight aneurysms: 7/10 treated using coiling with or without balloon-assistance, and 1/12 following intrasaccular flow disruption. None occurred following stent-assisted or flow diverter treatments. Compared to DSA both modalities showed high accuracy: AL-BB-MRI demonstrated accuracy of 0.73 (16/22, 95% CI: 0.5 to 0.89), sensitivity 0.62 (5/8, 95% CI: 0.24 to 0.91), and specificity: 0.79 (11/14, 95% CI: 0.49 to 0.95). TOF-MRA showed accuracy of 0.77 (17/22, 95% CI: 0.55 to 0.92), sensitivity 0.62 (5/8, 95% CI: 0.24 to 0.91) and specificity of 0.86 (12/14, 95% CI: 0.57 to 0.98). In nine cases of disagreement between AL-BB-MRI and TOF-MRA, AL-BB-MRI yielded three false positives and two false negatives, while TOF-MRA showed two false positives two false negatives.

**Table 5:** Accuracy measures of automatically labelled black-blood T1W-MRI and TOF-MRA compared to DSA

**Group**	**AL-BB-MRI**	**TOF-MRA**
Total (*n* = 22)	73%	77%
Flow diverter (*n* = 7)	100%	86%
Intrasaccular flow disruption (*n* = 5)	40%	80%
Stent-assisted coiling (*n* = 3)	67%	33%
Anterior cerebral artery (*n* = 8)	75%	62%
ICA (*n* = 6)	100%	67%
Posterior circulation (*n* = 8)	50%	100%

AL-BB-MRI: automatically labelled 3D black-blood T1W-MRI

**Fig. 3:**
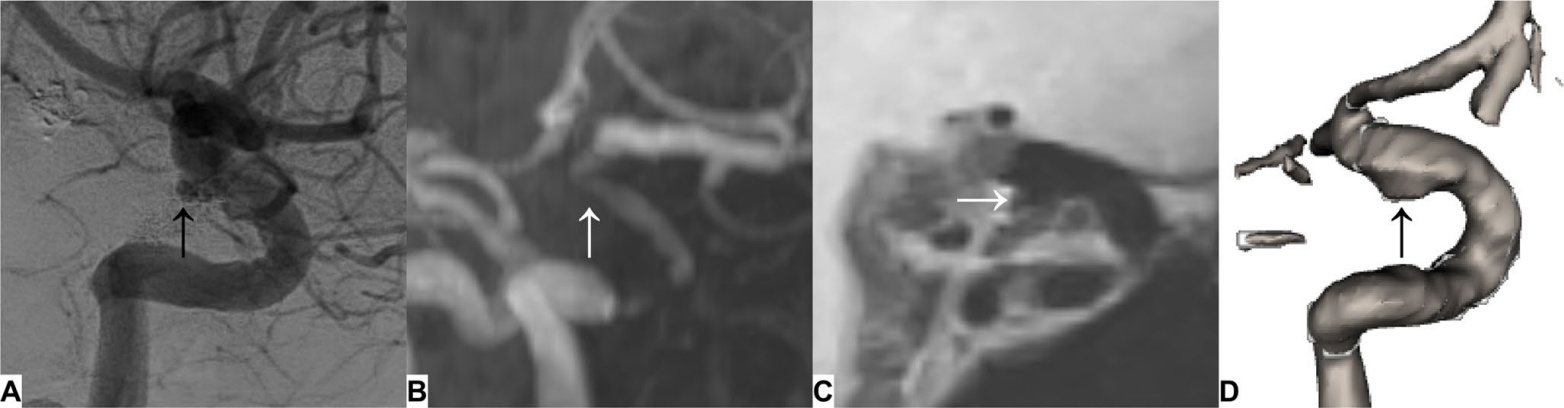
Lateral oblique projections of the left internal carotid artery six months after flow-diverter treatment with adjunctive coiling of a posterior communicating artery aneurysm. **A**, DSA shows a minor recurrence (black arrow). **B**, Time-of-flight MRA demonstrates typical signal loss at the flow-diverter site, obscuring the minor recurrence (white arrow). **C**, Sagittal reconstruction of 3d-T1w-black-blood MRI shows minimal contrast between the recurrence (white arrow) and the surrounding coils. **D**, Volume rendering of the segmentation results allows improved depiction of the minor recurrence (black arrow)

**Fig. 4:**
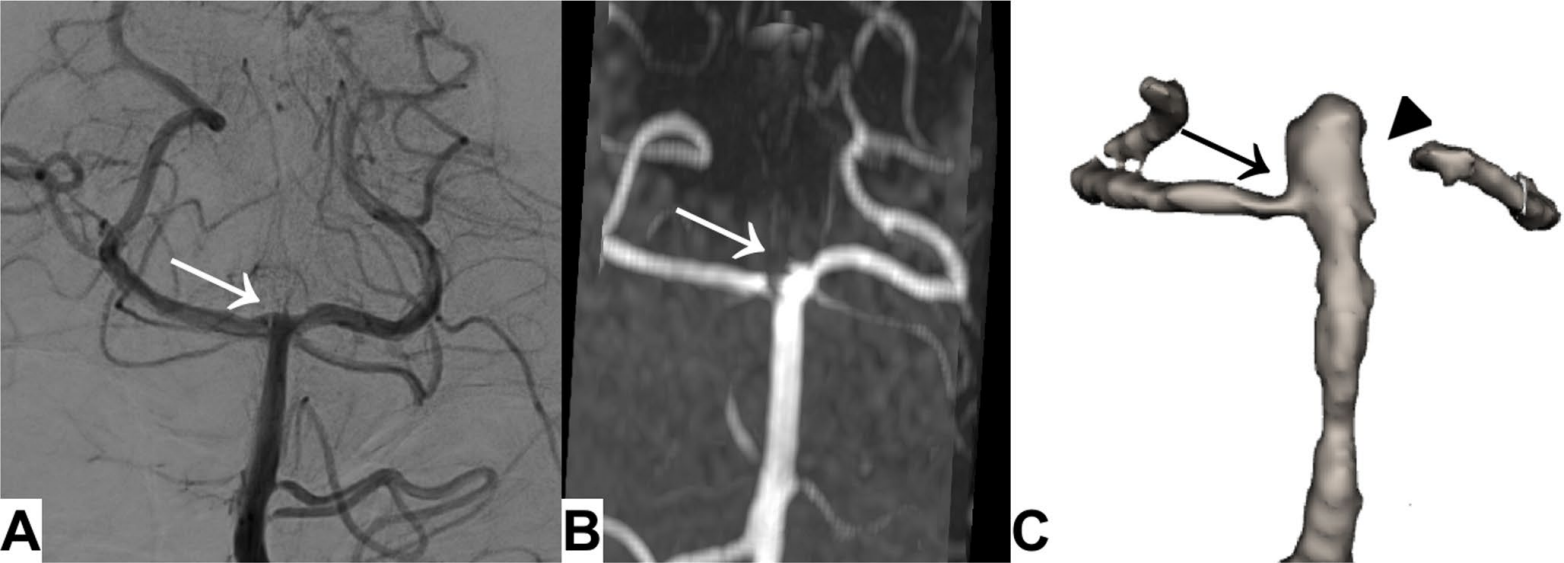
AP projections of the basilar artery following intrasaccular flow disruption of a tip of the basilar aneurysm. **A**-**B**, DSA and time of flight MRA show no recurrence (white arrow). **C**, 3D render of the segmentation results, suggesting a major recurrence (black arrow). The proximal posterior cerebral artery was also not depicted (black arrow head)

Subgroup analysis revealed superior AL-BB-MRI accuracy after flow diverter treatment (100% vs. 86%, Fig. 3) and stent assisted coiling (67% vs. 33%). However, AL-BB-MRI performed worse than TOF-MRA following intrasaccular flow disruption (40% vs. 80%), with AL-BB-MRI suggesting major recurrence in all cases (specificity 0.25 (1of4), Fig. 4). Both modalities adequately assessed anterior circulation aneurysms, while TOF-MRA was superior in the posterior circulation (Table 5).

## Discussion

We developed a CNN for cerebral vascular segmentation using high-resolution non-contrast 3D black-blood T1W-MRI, achieving excellent agreement with manual segmentation (DSC = 0.81). In a consecutive patient group, AL-BB-MRI allowed for aneurysm recurrence detection post-EVT with an accuracy comparable to that of TOF-MRA. We observed a high agreement of 78% between AL-BB-MRI and TOF-MRA, and a high accuracy of both modalities compared to DSA (73% and 77% for AL-BB-MRI and TOF-MRA, respectively).

Previous CNN vascular segmentation studies were based on TOF-MRA and reported DSCs of 0.8–0.9 [27–29]. Our model used 3D black-blood T1W-MRI achieving comparable accuracy (DSC = 0.81) to that of previous publications. However, other studies have described an effect of vessel size on segmentation accuracy, with a DSC of 0.41 for small-sized and 0.76 for middle- and large-sized vessel segments [29, 30]. Our anatomical subregion approach with complex network architecture improved small-vessel segmentation accuracy, though results remained highest in the ICA region and progressively decreased, with lowest accuracy in the M2 segment (Table 3).

Previous studies comparing TOF-MRA and DSA for post-EVT aneurysm recurrence reported TOF-MRA sensitivity/specificity of 0.86 [31], with interrater agreement improving from (κ = 0.28–0.5) on a three-point scale to κ = 0.58–0.62 when using a dichotomous scale [20, 32]. In our study, the readers showed consistent interpretation between AL-BB-MRI and TOF-MRA (mean intrarater agreement was 79%, interrater agreement 74% and 80% for the AL-BB-MRI and TOF-MRA, respectively). Consensus agreement between modalities was high reaching 78%, with Gwet’s AC1 of 0.69.

Recurrence evaluation was influenced by the effect of vessel size on AL-BB-MRI, with highest TOF-MRA agreement both in paraophthalmic aneurysms, and in the ICA territory overall. However, the vessel size effect alone cannot account for the superior performance in anterior communicating artery aneurysms compared to posterior circulation cases. This discrepancy may stem from two factors: First, the model may not have fully captured the distinct anatomical features of posterior circulation vasculature, despite using region-specific training and more complex network architectures. Second, the model likely benefited from the greater prevalence of anterior communicating aneurysm cases in our dataset, which may have enhanced its ability to learn characteristic signal patterns at the coil-vessel interface.

We collected data using clinically indicated DSA examinations, leading to a low number of available examinations and a selection bias with overrepresentation of device-assisted treatments (*n* = 12/22). In these cases, both AL-BB-MRI (0.73) and TOF-MRA (0.77) showed comparable accuracy to each other but lower than previously reported TOF-MRA values (0.86) [31]. We attribute this to the selection bias towards device-assisted treatments, which lead to signal loss in TOF-MRA [6, 7], and limited available DSA examinations rather than inherent differences favoring TOF-MRA or AL-BB-MRI. Following flow diverter treatment, neither DSA nor TOF-MRA detected any true recurrences, potentially due to device-related signal loss masking vessels in TOF-MRA. Similarly, AL-BB-MRI showed low specificity (0.25, 1/4) following intrasaccular flow disruption, likely because the CNN misinterpreted device-related signal loss as patent vasculature.

Beside widespread use of 3D black-blood T1W-MRI in vessel wall imaging, its diagnostic value in depicting vessel pathology was described previously, including applications in intracranial plaque characterization and detection of thrombosis of the venous sinuses [8, 33, 34]. However, the complex three-dimensional configuration of the vessels is difficult to appreciate when reporting using source images or multiplanar reconstructions. It is common radiological practice to use maximum intensity projections of TOF-MRA to provide an overview of the vascular tree. In black-blood MRI, minimum intensity projections could provide a vascular overview [34], but do not differentiate between arterial and venous structures. Therefore, segmentation of the arterial tree provides a distinct benefit over raw data or minimum intensity projections by generating a 3D vascular overview that selectively isolates arterial structures, eliminating venous contamination. Additionally, the machine learning-based algorithm can detect subtle signal intensity differences that may be difficult to appreciate visually in the source images, as demonstrated in Fig. 3.

Integrating 3D black-blood MRI into routine clinical follow-up is feasible. First, the necessary sequence is widely available on modern MRI scanners and is probably already implemented in most neurovascular centers. Second, its integration is relatively unproblematic from a safety and protocol standpoint, as it does not require contrast medium. The primary consideration is the additional examination time, which would increase the overall scan duration. Selective implementation in cases of anterior circulation aneurysms and following stent or flow diverter implantation, where its benefits may be most pronounced, may offer an alternative pragmatic strategy.

Study limitations include: First, the single-center design and homogeneous training data. Furthermore, although this study employed a widely adopted, vendor-supplied vessel wall imaging sequence [8], black-blood MRI techniques differ significantly between manufacturers. These vendor-specific variations in acquisition parameters and resulting image contrast could limit the generalizability of the model’s performance. Second, the training dataset was limited by the substantial time required for manual annotation (~ 70 min per scan), though this was mitigated by our patch-based CNN architecture that generated millions of training patches, yielding results consistent with prior studies [27, 28]. Third, clinical constraints led to a selection bias favoring device-assisted treatments in our DSA cohort, which may not represent the general post-EVT aneurysm population.

## Conclusions

Automated segmentation of cerebral vasculature using high-resolution non-contrast 3D black-blood MRI demonstrates promising performance in detection of aneurysm recurrences in the anterior circulation, suggesting its potential as an alternative or complementary tool for post-EVT follow-up, particularly after flow diverter treatment. However, performance was limited for posterior circulation aneurysms and intrasaccular flow disruption cases (Level of Evidence: 5B).

## Supplementary Information

Below is the link to the electronic supplementary material.

Supplementary Material 1 (PDF 232 KB)

Supplementary Material 2 (XLSX 13.8 KB)

## Data Availability

Anonymized data enabling replicating of the results are available in the supplementary material (reference to xlsx file).
