# Supplementary material for: Deep learning-based vessel segmentation in non-contrast black-blood MRI for treated aneurysm follow-up: a comparative study with TOF-MRA and DSA
Source: Neuroradiology. 2026 Mar 6;68(4):987–96. doi: 10.1007/s00234-026-03936-7 (PMC13139250; doi:10.1007/s00234-026-03936-7)
Supplement: Supplementary file 1 — Supplementary Material 1 (PDF 232 KB) [file 234_2026_3936_MOESM1_ESM.pdf]

Online Supplemental Data

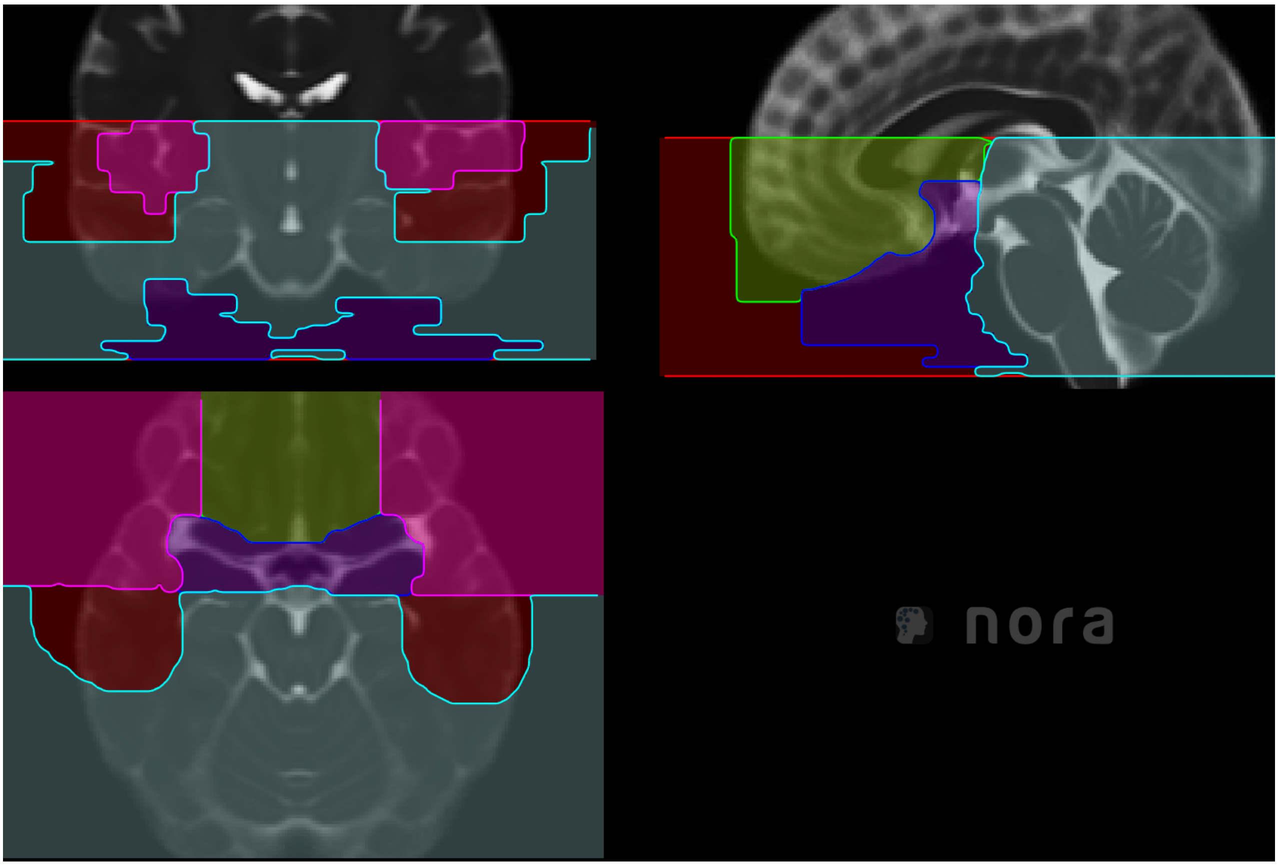

Figure S1: Binary masks of the 4 subregional models in MNI space.

Table S1: Patient characteristics in all datasets.

Variable	Training (22 patients 27 aneurysms)	Validation (4 patients 4 aneurysms)	Testing (5 patients 6 aneurysms)	Recurrence (84 patients 90 aneurysms) *
Age	55.6 (±13.2)	46.8 (±12.3)	53.5 (±5)	54.3 (±11.7)
No. of female patients	16 (59%)	0 (0%)	2 (33%)	63 (70%)
Aneurysm Location				
Basilar artery	0	0	1 (17%)	2 (2%)
Anterior communicating artery	6 (22%)	1 (25%)	4 (67%)	39 (43%)
Posterior communicating artery	2 (7%)	1 (25%)	1 (17%)	12 (13%)
Basilar artery	3 (11%)	0	0	12 (13%)
Pericallosal artery	1 (4%)	1 (25%)	0	0
Internal carotid artery	8 (30%)	0	0	7 (8%)
Middle cerebral Artery	1 (4%)	0	0	1 (1%)
Basilar artery	1 (4%)	0	0	1 (1%)
Vertebral artery	2 (7%)	0	0	3 (3%)
Anterior choroidal artery	2 (7%)	0	0	6 (7%)
Internal carotid artery	1 (4%)	0	0	1 (1%)
Vertebral artery	0	1 (25%)	0	1 (1%)
Basilar artery	0	0	0	1 (1%)
A1/A2 segments	0	0	0	1 (1%)
A1/A2 segments	0	0	0	1 (1%)
Internal carotid artery	0	0	0	2 (2%)
Clinical Data				
Presentation: Incidental	19 (70%)	1 (25%)	5 (83%)	42 (47%)
Presentation: Ruptured	8 (30%)	3 (75%)	1 (17%)	48 (53%)

Treatment and Results				
Coiling	22 (81%)	4 (100%)	5 (83%)	78 (87%)
Balloon-assisted Coiling	16 (59%)	2 (50%)	5 (83%)	59 (66%)
Stent-assisted Coiling	3 (11%)	1 (25%)	0	10 (11%)
Flow diverter	7 (26%)	0	1 (17%)	10 (11%)
Intrasaccular Flow Disruption	1 (4%)	0	0	7 (8%)
Complete Occlusion	6 (22%)	4 (100%)	5 (83%)	51 (57%)
Neck Remnant	11 (41%)	0	0	23 (26%)
Aneurysm Rest	10 (37%)	0	1 (17%)	16 (18%)
No / Minor Recurrence	20 (74%)	4 (100%)	6 (100%)	76 (84%)
Major Recurrence	7 (26%)	0	0	14 (16%)

Age is presented as mean ±SD. Categorical variables are presented as number with percentages in parenthesis. *This column includes patients from the testing group.

Table S2: All metrics of the final models

Model	Threshold	Dice	Hausdorff (100%)	Hausdorff (95%)	Surface avg dist 1	Surface avg dist 2	Surface dice (1mm)	Surface overlap 1 (1mm)	Surface overlap 2 (1mm)
A2-segment subregional model	60	0.67	19.92	4.83	0.41	0.65	0.88	0.89	0.88
ICA/A1-segment subregional model	70	0.85	15.67	3.99	0.21	0.68	0.96	0.97	0.96
M2-segment subregional model	70	0.58	13.95	6.56	0.41	1.76	0.82	0.90	0.80
Posterior circulation subregional model	70	0.73	28.37	16.67	0.65	1.48	0.88	0.84	0.94
Ensemble model	60	0.81	31.15	5.82	0.30	1.18	0.91	0.93	0.89
